# Prevalence, risk factors, and virulence genes of *Helicobacter pylori* among dyspeptic patients in two different gastric cancer risk regions of Thailand

**DOI:** 10.1371/journal.pone.0187113

**Published:** 2017-10-30

**Authors:** Phawinee Subsomwong, Muhammad Miftahussurur, Tomohisa Uchida, Ratha-korn Vilaichone, Thawee Ratanachu-ek, Varocha Mahachai, Yoshio Yamaoka

**Affiliations:** 1 Department of Environmental and Preventive Medicine, Oita University Faculty of Medicine, Yufu, Japan; 2 Gastroentero-Hepatology Division, Department of Internal Medicine, Faculty of Medicine-Dr. Soetomo Teaching Hospital-Institute of Tropical Disease, Universitas Airlangga, Surabaya, Indonesia; 3 Department of Gastroenterology and Hepatology, Baylor College of Medicine and Michael DeBakey Veterans Affairs Medical Center, Houston, Texas, United States of America; 4 Department of Molecular Pathology, Faculty of Medicine, Oita University, Hasama-machi, Yufu-City, Oita, Japan; 5 Gastroenterology Unit, Department of Medicine, Thammasat University Hospital, Pathum Thani, Thailand; 6 Department of Surgery, Rajavithi Hospital, Bangkok, Thailand; 7 GI and Liver Center, Bangkok Medical Center, Bangkok, Thailand; University Hospital Llandough, UNITED KINGDOM

## Abstract

Gastric cancer risk is varied among different regions of Thailand. We examined the characteristics of *Helicobacter pylori* infection in two regions of Thailand. The *H*. *pylori* status of 273 dyspeptic patients (136 from the South and 137 from the North; a low and high incidence of gastric cancer region, respectively) was evaluated, and virulence genotypes (*cagA*, *vacA*, *hrgA* and *jhp0562*-positive/*β-(1*,*3)galT*) were determined. The overall *H*. *pylori* infection rate was 34.1% (93/273). The prevalence was higher in the North than in the South (50.4% vs. 17.6%, P <0.001) and was significantly higher among individuals with the following characteristics: low income, birthplace in the Northeast or North regions, agricultural employment, or consumption of alcohol or unboiling water. Among these socio-demographic determinants, region was an independent risk factor for *H*. *pylori* infection (odds ratio = 6.37). Patients including both *H*. *pylori* infected and uninfected cases who lived in the North had significantly more severe histological scores than those in the South. In contrast, among *H*. *pylori*-positive cases, patients in the South had significantly more severe histological scores than those in the North. Of the 74 strains cultured, 56.8% carried Western-type *cagA*, with a higher proportion in the South than in the North (76.2% vs. 49.1%, P = 0.05). In disagreement with the current consensus, patients infected with the Western-type *cagA* strains had more severe inflammation scores in the antrum than those infected with the East Asian-type *cagA* strains (P = 0.027). Moreover, Western-type *cagA* strains induced more severe histological scores in patients from the South than those of either genotype from the North. Other virulence genes had no influence on histological scores. The incidence of gastric cancer in Thailand was different among regions and corresponded to differences in the prevalence of *H*. *pylori* infection. More careful follow-up for patients in the South will be required, even if they are infected with *H*. *pylori* carrying Western-type *cagA*.

## Introduction

For more than a century, it was believed that the presence of bacteria in the human stomach came from digested food contaminants, until the identification of a gram-negative bacteria, *Helicobacter pylori*, which is a causative agent of various severe gastroduodenal diseases, including gastric cancer [1]. Accordingly, the risk of developing gastric cancer is reduced following *H*. *pylori* eradication, and eradication should be performed before there is irreversible damage done to the patient [2].

Several environmental factors are associated with an increased risk of *H*. *pylori* infection, and these factors are found among those living in developing countries and in lower socioeconomic groups in the developed world, probably due to poor hygiene standards and crowded households [3]. A previous study showed that improved hygiene conditions have association with the prevalence of *H*. *pylori* infection in many parts of North America and Europe [4]. *H*. *pylori* infection rates also decreased in those born after 1950, simultaneously with a rapid change in sanitary conditions and in the standard of living. These changes include the development of a clean public water systems in Japan following World War II [5]. In addition to bacterial and host factors, there is a positive correlation between gastric cancer risk and smoking habits [6] and alcohol consumption [7], in addition to the fermented food [8], salt [9], and nitrates [10, 11].

It is believed that multistep and multifactorial precancerous processes that are associated with persistent chronic mucosal inflammation and the direct action of *H*. *pylori* virulence factors determine the severity of disease and long-term patient outcome [12]. It has been reported that individuals infected with *cagA*-positive *H*. *pylori* strains have an increased risk of peptic ulcer disease, atrophic gastritis, intestinal metaplasia, and gastric cancer, compared to individuals who are infected with *cagA*-negative strains [13, 14]. The second repeat of the C-terminal region of CagA sequences has a difference between East Asian-type CagA and Western-type CagA. East Asian-type CagA has a higher binding affinity for the Src homology-2 domain-containing phosphatase 2 (SHP2), resulting in a greater risk of peptic ulcer development and/or gastric cancer when compared to its Western counterpart [15–18]. In addition, examination of the sequence of the 300 bp region upstream of the first EPIYA (Glu-Pro-Ile-Tyr-Ala) motif in the C-terminal region of CagA, denoted hereafter as the pre-EPIYA region, revealed that a 39-bp deletion is present in most strains isolated from East Asia, but is absent from most strains from Western countries (denoted as the no deletion type) [19]. A previous study also showed that the EPIYA sequence in the EPIYA-B motif is more highly associated with gastric cancer than the EPIYT sequence is, which might be due to differences in its affinity for phosphoinositol 3-kinase and in its induction of protein kinase B [20]. Finally, a conserved sequence motif of 16 amino acids (FPLXRXXXVXDLSKVG) in the C-terminal region of CagA is a prerequisite for the CagA-SHP-2 interaction, and 11 of the 16 amino acids in the CagA multimerization (CM) motif are well conserved between Western- (containing multiple CM motifs) and East Asian-type (containing a single CM motif) CagA strains [21]; therefore, the type and number of CM motifs may influence the potential of individual CagA proteins to multimerize in host cells [22].

*cagA* status is linked to the *vacA* genotype, which causes variations in the vacuolating activity of different *H*. *pylori* strains. The s1m1i1-type strains produce vacuolating toxin (VacA) and are more likely to be associated with gastric cancer than the s2m2i2 type, which is a non-vacuolating type [23]. Other regions, such as a deletion (d) [24, 25] and a polymorphic site in the 3′ end of *vacA*, denoted as the c region, have been reported to be better predictors of disease severity than either the s or m region [26]. Additionally, there have been one report that the presence of *hrgA* is increased among gastric cancer patients in East Asia [27], although the subsequent studies from other group could not confirm the association [28, 29]. There were also several reports that the presence of *jhp0562* and *β-(1*,*3)galT* is associated with the development of peptic ulcers [30, 31].

Thailand is a country in Southeast Asia with an *H*. *pylori* infection rate ranging from 54.1% to 76.1% [32]; however, the age-standardized incidence rate (ASR) of gastric cancer has been reported to be 3.1/100,000, which is relatively low among Asian countries (data available from the International Agency for Research on Cancer; GLOBOCAN2012, http://globocan.iarc.fr/). Importantly, gastric cancer risk in Thailand is varied among different regions. The North has the highest incidence rate (ASR: 7.9 for males and 5.2 for females); in contrast, the South has the lowest incidence rate (ASR: 2.0 for males and 1.4 for females) in Chiang Mai and Songkhla province, respectively [33]. It is important to analyze factors affecting the different gastric cancer risk between these two regions. In this study, we examined the prevalence of *H*. *pylori* infection and also identified and analyzed environmental factors in both regions. Furthermore, we collected *H*. *pylori* strains from both regions and evaluated the virulence factors of those strains.

## Materials and methods

### Study population

**Fig 1:**
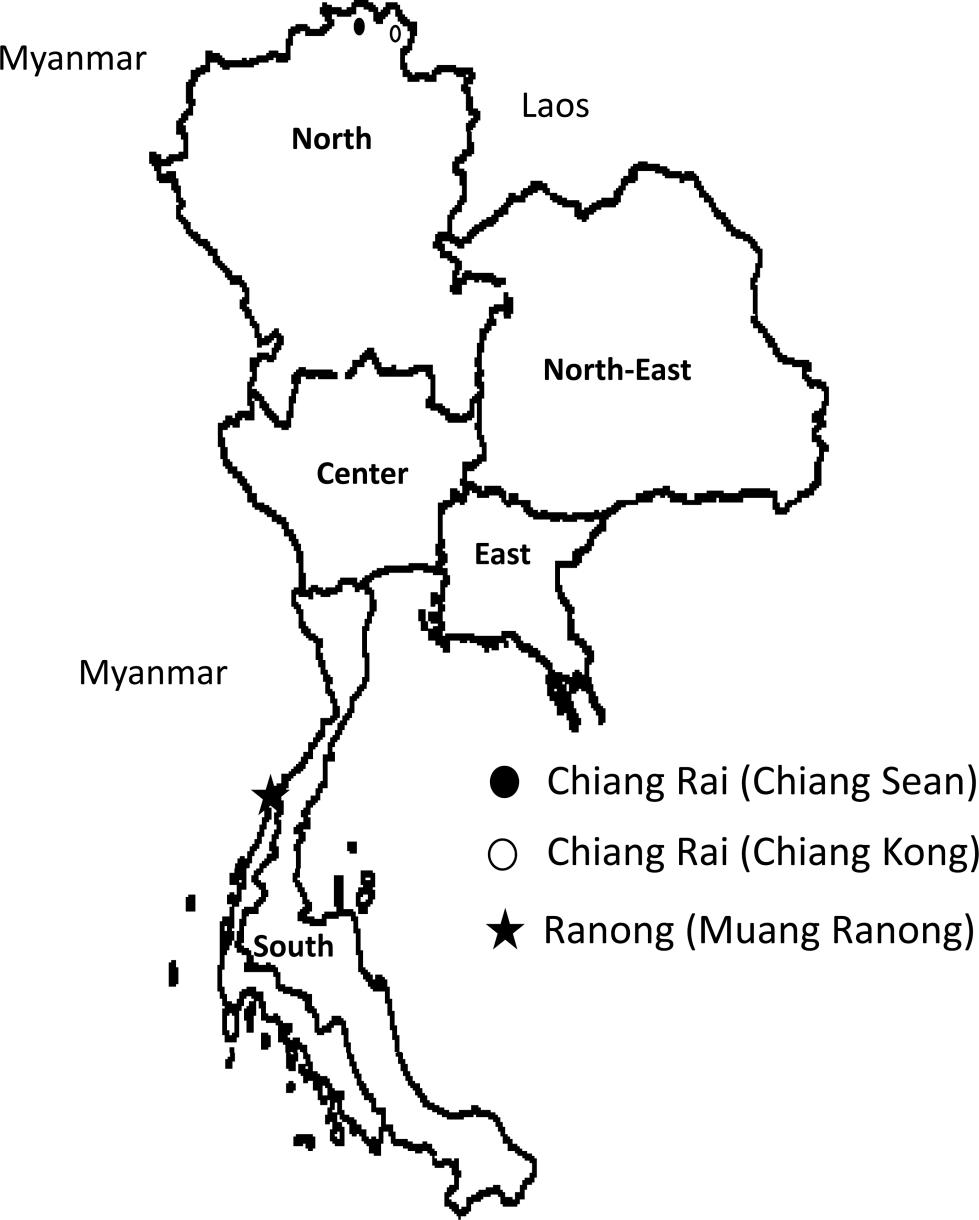
The locations in this study. Ranong province (black star; Muang Ranong) and Chiang Rai province (black circle: Chiang Saen; white circle: Chiang Kong) are located in Southern and Northern Thailand, respectively. Ranong province is along the West coast of Andaman Sea and share a border with Myanmar, while Chiang Rai is mountainous area that have Mekong River separating Thailand with Myanmar and Laos.

We performed a cross sectional study in dyspeptic patients who were living in Muang Ranong city, Ranong province, which is in the South of Thailand, on August 28, 2015. From January 19–20, 2016, we also recruited dyspeptic patients from two cities in the Chiang Rai province, which is in the North: Chiang Kong and Chiang Saen (Fig 1). The project was announced by local hospital officers and all the participants were living in the community. Questionnaire information was gathered before an endoscopy. Experienced endoscopists collected three gastric biopsy specimens during each endoscopy session: two samples from the antrum, approximately 2–3 cm from the pyloric ring, and one sample from the greater curvature of the corpus. To minimize potential bias, we used the same experienced pathologist (TU), who has also performed histological evaluations of patients from Nepal, Myanmar, Vietnam, Bhutan, the Dominican Republic, and Indonesia [34–40]. Biopsy specimens for bacterial culture were immediately placed in transport media and were put in a mobile freezer at -20°C and were transported to Thammasat University Hospital, Thailand within a day of collection and were kept at -80°C. Then, we transported the biopsy specimens with dry ice to the Central Laboratory in Oita University Faculty of Medicine, Japan. All biopsy specimens could be kept frozen during the transportation and were directed to culture within 12 hours upon arrival of the specimens. Overall, although the total duration time from collecting biopsy specimens in Thailand to starting culture in Japan varied (average 20 days), duration time with the temperature above -80°C was less than 24 hours. Two antral specimens were used for the *H*. *pylori* culture and histological examinations. One corporal specimen was used for histological examinations. Peptic ulcers were identified through endoscopy. Written informed consent was obtained from all participants, and the study protocol was approved by the Ethics Committee of Faculty of Medicine, Thammasat University (Pathum Thani, Thailand) and Oita University Faculty of Medicine (Yufu, Japan).

### *H*. *pylori* infection status

*H*. *pylori* infection status was diagnosed based on culture and histology, which was confirmed by immunohistochemistry (IHC), as the tests could directly check for the presence of *H*. *pylori*. For the *H*. *pylori* culture, one antral biopsy specimen was homogenized and directly inoculated onto *H*. *pylori* selective media (Nissui Pharmaceutical co., LTD, Tokyo, Japan). The plates were incubated for up to 7 days at 37°C under microaerophilic conditions (10% O_2_, 5% CO_2_ and 85% N_2_). The *H*. *pylori* like colony were sub-cultured on Mueller Hinton II Agar medium (Becton Dickinson, NJ, USA) supplemented with 10% horse blood without antibiotics (Nippon Biotest Laboratories Inc., Tokyo, Japan) and confirmation by gram staining (spiral red rod)[41]. Isolated strains were stored at -80°C in Brucella Broth (Becton, Dickinson and Company, Sparks, MD, USA) containing 10% dimethyl sulfoxide and 10% horse serum.

For histology, one antral and one corporal specimen from each patient was examined. Biopsy materials were fixed in 10% buffered formalin and embedded in paraffin. Serial sections were stained with hematoxylin and eosin, as well as with May and Giemsa stains. The degree of inflammation, neutrophil activity, atrophy, intestinal metaplasia, and bacterial density were classified into four grades, in accordance with the updated Sydney system: 0, ‘normal’; 1, ‘mild’; 2, ‘moderate’; and 3, ‘marked’ [42]. Samples with a grade of 1 or higher of atrophy were considered atrophy-positive. In addition, the gastritis stage was assessed based on the topographic location (antrum or corpus) of atrophy, in accordance with the Operative Link on Gastritis Assessment (OLGA) system [43].

IHC for an anti-*H*. *pylori* antibody (Dako, Glostrup, Denmark) was performed, as previously described [44].

### *H*. *pylori* genotyping

DNA was extracted from the cultured strains using the QIAamp DNA Mini Kit (QIAGEN, Valencia, CA, USA), in accordance with the manufacturer’s directions. The primer list is shown in S1 Table. The presence of *vacA* (s, m, i, d, and c regions), *hrgA*, *jhp0562*, and *β-(1*,*3)galT* were determined based on polymerase chain reaction (PCR) product size, as described previously [19, 23, 25, 26, 45–48], using the Microchip Electrophoresis system for DNA/RNA Analysis MCE® -202 Multina (Shimazu, Kyoto, Japan). *cagA* genotypes (positive/negative, EPIYA repeat region, CM motif, and pre-EPIYA) were determined by PCR amplification-direct sequencing using the ABI 3100 Genetic Analyzer DNA Sequencer.

### Interluekin-8 production from gastric epithelial cell infected with *H*. *pylori* strains

The human adenocarcinoma gastric cell line (AGS) were seeded into 24-well plates at a density of 5×10^4^ cells per well and incubated in RPMI 1640 (Nacalai Tesque, Inc., Kyoto, Japan) supplement with 10% heat-inactivated fetal bovine serum (Gibco, Grand Island, NY, USA) at 37°C in humidified incubator containing 5% CO_2_ for 2 days before infected with *H*. *pylori* strains. We selected 20 strains; 5 from the North with Western-type *cagA*, 5 from the North with East-Asian-type *cagA*, 5 from the South with Western-type *cagA*, 5 from the South with East-Asian-type *cagA*. As control, we used 2 reference strains (ATCC26695 with Western-type *cagA* and TN2 with East-Asian-type *cagA*) and mock-infection. All the *H*. *pylori* strains were cultured on Brucella agar supplement with 10% horse serum plate for 24 hours at 37°C before infection (Becton, Dickson and company, Sparks, MD, USA). The AGS cells were co-cultured with *H*. *pylori* at an MOI of 100 and incubated at 37°C in humidified incubator containing 5% CO_2_ for 24 hours. The medium was removed for measuring the IL-8 production by using Human IL-8 ELISA kit (eBioscience, Inc., Science Center Dr., San Diego, CA, USA) following the manufacturer’s instruction. All samples were measured in duplicate in three independent experiments.

### Statistical analysis

Discrete variables were tested using a chi-square test (the prevalence of *H*. *pylori*, demographics and sanitation status, and genotypes vs. regions or diagnosis). The difference of histologic score and IL-8 levels between genotypes were tested using the Mann-Whitney *U* test. A multivariate logistic regression model was used to calculate the odds ratios (OR) of the clinical outcomes. All determinants with P values less than 0.10 were entered together into the full logistic regression model, and the model was reduced by excluding variables with P values greater than 0.10. OR and a 95% confidence interval (CI) was used to estimate the risk. A P value less than 0.05 was accepted as statistically significant. The SPSS statistical software package version 18.0 (SPSS, Inc., Chicago, IL) was used for all statistical analyses.

## Results

### Prevalence of *H*. *pylori* infection

We initially registered 340 dyspeptic patients (170 each from the North and South). Among them a total of 306 patients went to the hospital on the scheduled day and were recruited consecutively: Ranong in the South (n = 158) and Chiang Kong (n = 102) and Chiang Saen (n = 46) in the North. We excluded 33 patients: 8 patients had missing histology information, and 25 patients had histories of *H*. *pylori* eradication. Therefore, the total study population was 273 patients (265 with gastritis and 8 with peptic ulcers). The population consisted of 136 patients from the South (93 females and 43 males; mean age of 52.0 ± 14.7 years; age range of 15–82 years) and 137 patients from the North (85 females and 52 males; mean age of 56.4 ± 13.6 years; age range of 21–88 years).

The prevalence of *H*. *pylori* infection was the highest when samples were examined with a histological test (32.3%, P <0.001), while it was 27.1% by bacterial culture (Table 1). We determined that the patient was *H*. *pylori*-positive if culture and/or histology confirmed by IHC had positive results; the final prevalence was determined to be 34.1%. The prevalence in the North was higher than that in the South (50.4% vs. 17.6%, P <0.001) (Table 1). As there was no difference in the prevalence between the two cities in the North, Chiang Kong and Chiang Saen (50.5% vs. 50.0%, P = 0.95), we combined both cities in the subsequent analyses.

**Table 1:** Prevalence of *H*. *pylori* infection in each diagnostic test (%).

Methods	Region		Age					Total
	North	South	≤29	30–39	40–49	50–59	≥60	
N	137	136	19	23	53	83	95	273
Culture	53 (38.7)	21 (15.4)	5 (26.3)	4 (17.4)	19 (35.8)	25 (30.1)	21 (22.1)	74 (27.1)
Histology confirmed by immunohistochemistry	66 (48.2)	22 (16.2)	6 (31.6)	3 (13.0)	22 (41.5)	32 (38.6)	25 (26.3)	88 (32.2)
Histology confirmed by IHC and/or culture	69 (50.4)	24 (17.6)	6 (31.6)	5 (21.7)	23 (43.4)	34 (41.0)	25 (26.3)	93 (34.1)

### Demographics, sanitation, *H*. *pylori*, and region

The OR was calculated for *H*. *pylori* infection rate (Table 2 and S2 Table). Subjects who were born in the Northeast and North regions had significantly higher *H*. *pylori* infection rate than those born in the South (OR = 10.5 and OR = 5.34, respectively). The prevalence of *H*. *pylori* infection among subjects who had low income or consumed boiling water or alcohol consumption was significantly higher than the prevalence among subjects who had high economic status (OR = 3.00), boiling their drinking water (OR = 1.93), or did not consume alcohol (OR = 2.21), respectively. Agriculture was an occupation with the highest risk of *H*. *pylori* infection (OR = 3.92). In contrast, there were no statistically significant relationships between *H*. *pylori* infection rate and gender, ethnicity, marital status, body mass index, consumption of fried and fermented diet, or smoking habits. When we entered all determinants with P values of less than 0.10 by bivariate analysis (age, sex, region, income status, source of drinking water, occupation, and alcohol consumption) into the full logistic regression model, subjects who were living in the North had a higher risk for *H*. *pylori* infection than did those in the South (OR = 6.37 [CI 2.91–13.96], P <0.001)

**Table 2:** Association of demographics and sanitation levels with *H*. *pylori* infection status.

Variable	*H*. *pylori*-positive/total number (%)	Crude OR	95% CI for OR	P
Birthplace				
Central	0/1 (0.0)	0.0	0.00	0.99
East	1/2 (50.0)	5.25	0.32–87.44	0.25
Lao	4/8 (50.0)	5.25	1.21–22.74	0.03
North	62/123 (50.4)	5.34	2.94–9.67	0.001
Northeast	6/9 (66.7)	10.50	2.42–45.49	0.002
South	20/125 (16.0)	1.00		
Others	0/5 (0.0)	0.0	0.00	0.99
Social economic status				
No income	14/43 (32.6)	2.03	0.63–6.50	0.24
<5,000 THB (142.5 USD)	30/76 (39.5)	2.74	0.93–8.05	0.07
5,000–10,000 THB	30/72 (41.7)	3.00	1.02–8.85	0.047
10,000–20,000 THB	13/50 (26.0)	1.48	0.46–4.72	0.51
30,000–50,000 THB	5/26 (19.2)	1.00		
50,000–100,000 THB	0/3 (0.0)	0.00	0.00	0.99
Source of drinking water				
Boiling	45/157 (28.7)	1.00		
Unboiling	48/110 (43.6)	1.93	1.16–3.21	0.012
Alcohol consumption				
No	43/161 (26.7)	1.00		
Yes	50/112 (44.6)	2.21	1.33–3.69	0.002

P <0.05, Chi-square, estimate risk by OR and 95% CI

Table 3 shows the demographics and sanitation status in both regions. Individuals from the North had a significantly higher mean age and lower economic status than individuals from the South (P = 0.01 and P <0.001, respectively). There were significant differences in ethnic composition and obesity of subjects in both regions. Unboiling water and alcohol consumption was higher among subjects who were living in the North than among those from the South (for both, P <0.001). There was no statistical difference seen with differing gender, marital status, consumption of fried or fermented food, or smoking habits in either region.

**Table 3:** Demographics, sanitation, and regions.

Variable	North (%)	South (%)	P
N	137	136	
Mean age ± SD	56.4 ± 13.61	52.0 ± 14.66	0.01
Female	85 (62.0)	93 (68.4)	0.27
Thai ethnic	111 (81.0)	135 (99.3)	<0.001
Income ≤ 10,000 THB	118 (86.1)	73 (53.7)	<0.001
Agricultural worker	70 (51.1)	11 (8.1)	0.16
Married	113 (82.5)	107 (78.7)	0.25
Obesity	32 (23.4)	54 (39.7)	0.004
Unboiling water	84 (61.3)	26 (19.1)	<0.001
Fried food consumption	73 (53.3)	73 (53.7)	0.95
Fermented food consumption	50 (36.5)	47 (34.6)	0.74
Smokers	39 (28.5)	35 (25.7)	0.61
Alcohol consumption	76 (55.5)	36 (26.5)	<0.001

P <0.05, for categorical variable by Chi-square, for mean age by t-test.

### Gastric mucosal status and region

First, we examined the histology of patients including both *H*. *pylori* positive and negative cases. Histological findings scored for atrophy showed that 184 patients’ antrum biopsies (67.4%) were grade 0; 77 (28.2%) were grade 1, 11 (4.0%) were grade 2, and none were grade 3. Additionally, 243 patients’ corpus biopsies (89.0%) were grade 0, 25 (9.2%) were grade 1, 3 (1.1%) were grade 2, and only 2 (0.7%) were grade 3. When the biopsies with grade 1 or more were considered to be atrophy-positive, 88 patients (32.4%) had antral atrophy, and 30 patients (11.0%) had corporal atrophy. OLGA scores showed 179 biopsies (65.6%) were stage 0, 79 (29.0%) were stage I, 11 (4.0%) were stage II and only 3 (1.1%) were stage III, while stage IV was not found.

Histological scores broken down by region (including both *H*. *pylori* positive and negative patients) are shown in S3 Table and S1 Fig. Patients who were living in the North had significantly higher histological scores in the antrum and OLGA scores than those from the South had. Moreover, activity, inflammation, and bacterial density scores in the corpus were significantly higher in patients who were living in the North than in those living in the South.

Next, we took the *H*. *pylori* infection into account. As expected, all histological scores, aside from intestinal metaplasia, were significantly higher in patients positive for *H*. *pylori* than in those who were negative for *H*. *pylori* (S3 Table and S2 Fig). There was a high prevalence of *H*. *pylori* infection in the North and low prevalence in the South, suggesting that *H*. *pylori* infection could partially explain the different incidence rates of gastric cancer among individuals from various regions in Thailand.

**Fig 2:**
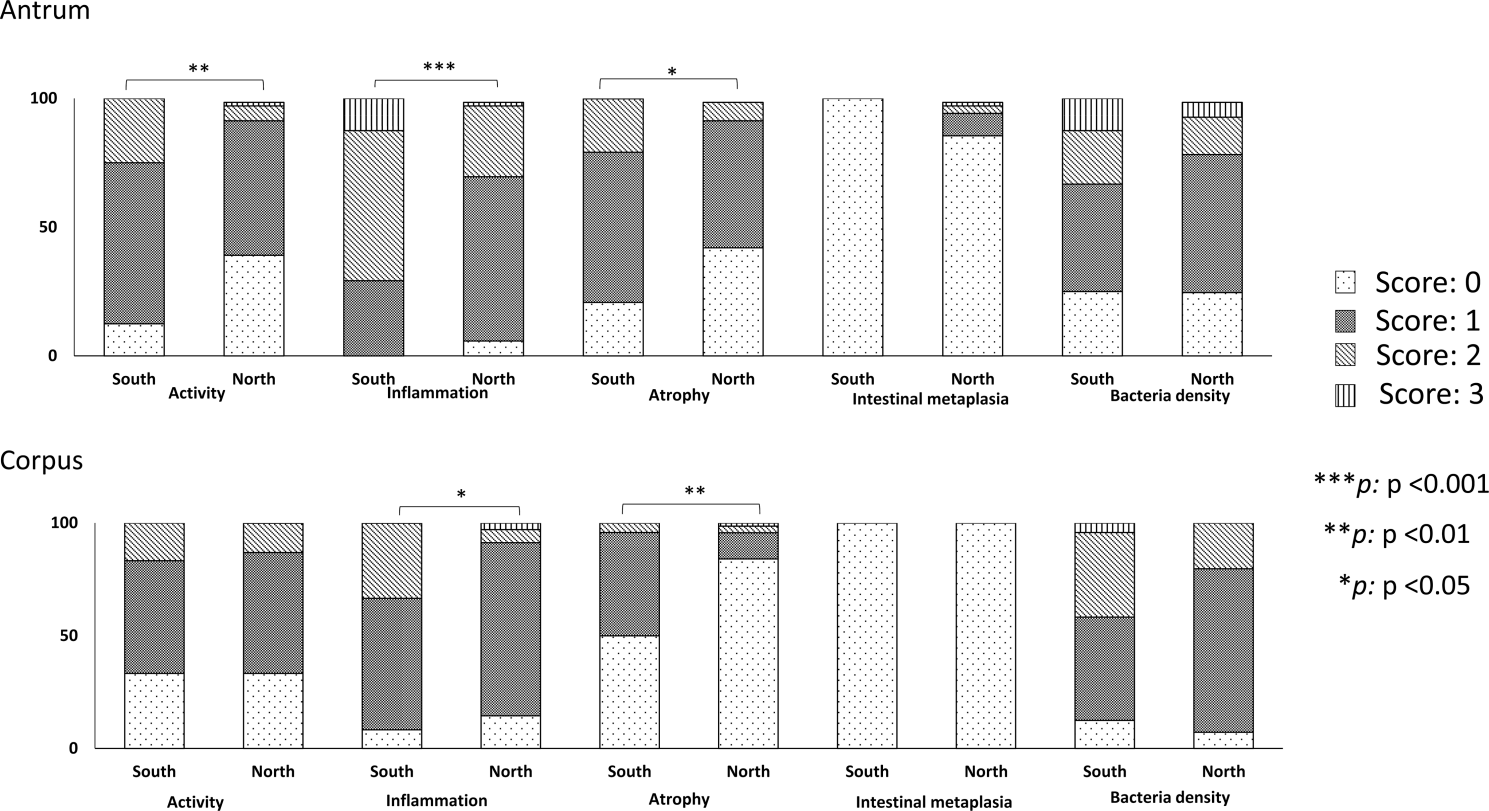
Histological scores of *H*. *pylori*-infected groups by region. Patients who were living in the South (n = 24) had significantly higher activity in the antrum, in addition to higher inflammation and atrophy scores in both the antrum and corpus, than did those who were living in the North (n = 69)(P <0.05, Mann-Whitney U test).

In contrast with our expectations for patients with an *H*. *pylori* infection, infected patients living in the South had significantly more severe activity, inflammation, and atrophy scores in the antrum than did patients in the North (mean [median]: 1.13 [1] vs. 0.70 [1], P = 0.004, 1.83 [1] vs. 1.25 [1], P < 0.001, 1.0 [1] vs. 0.65 [1], P = 0.024, respectively). Similar results were also found for inflammation and atrophy scores in the corpus (1.25 [1] vs. 0.97 [1], P = 0.018 and 0.54 [0.5] vs. 0.22 [0], P = 0.002) (Table 4 and Fig 2).

**Table 4:** Histological scores by *H*. *pylori* infection and *cagA* genotype.

Histological parameters	*H*. *pylori*-positive		p	*cagA* genotype		p	Western-type *cagA*		p
	North	South		Western	East Asian		North	South	
N	69	24		42	30		26	16	
**Antrum**									
Activity	0.70 (1)	1.13 (1)	0.004	0.93 (1)	0.77 (1)	0.333	0.72 (1)	1.25 (1)	0.015
Inflammation	1.25 (1)	1.83 (1)	<0.001	1.63 (2)	1.33 (1)	0.027	1.44 (1)	1.94 (2)	0.026
Atrophy	0.65 (1)	1.00 (1)	0.024	0.85 (1)	0.70 (1)	0.371	0.72 (1)	1.06 (1)	0.120
Intestinal metaplasia	0.19 (0)	0.00 (1)	0.062	0.07 (0)	0.23 (0)	0.368	0.12 (0)	0.00 (0)	0.155
Bacterial density	1.01 (1)	1.21 (1)	0.422	1.27 (1)	1.07 (1)	0.240	1.28 (1)	1.25 (1)	0.816
**Corpus**									
Activity	0.80 (1)	0.83 (1)	0.846	0.79 (1)	0.83 (1)	0.732	0.69 (1)	0.94 (1)	0.305
Inflammation	0.97 (1)	1.25 (1)	0.018	1.04 (1)	1.07 (1)	0.653	0.96 (1)	1.19 (1)	0.141
Atrophy	0.22 (0)	0.54 (0.5)	0.002	0.31 (0)	0.2 (0)	0.771	0.19 (0)	0.50 (0)	0.013
Intestinal metaplasia	0.00 (0)	0.00 (0)	1.000	0.02 (0)	0.0 (0)	0.398	0.04 (0)	0.00 (0)	0.433
Bacterial density	1.13 (1)	1.33 (1)	0.153	1.21 (1)	1.2 (1)	0.984	1.08 (1)	1.44 (1.5)	0.094
**OLGA score**	0.75 (1)	1.04 (1)	0.052	0.95 (1)	0.77 (1)	0.357	0.84 (1)	1.13 (1)	0.183

P <0.05, Mann-Whitney U test

### *H*. *pylori* genotype and gastric mucosal status

We focused on bacterial factors in order to determine whether there were different genotypes in the two regions. We were able to obtain 74 strains by bacterial culture: 53 from the North and 21 from the South (Table 5). In total, all strains contained *cagA*, and the Western-type *cagA* was predominant (56.8% of all strains). One strain each of AB, ABB, and ABBB type was regarded as Western-type *cagA* based on the sequence similarity of B segments with Western-type *cagA*. Overall, 40.5% of strains possessed the East Asian-type *cagA*. Two strains were *cagA*-positive by PCR; however, the *cagA* genotype for these two strains was undetermined by sequencing, since the sequence primer pair did not amplify *cagA*.

**Table 5:** Distribution of *H*. *pylori* genotypes.

Gene	Genotype	Region (%)		
		North (N = 53)	South (N = 21)	Total (N = 74)
cagA*	Western-type	26 (49.1)	16 (76.2)	42 (56.8)
	- AB	1 (3.8)	0 (0.0)	1 (2.4)
	- ABB	0 (0.0)	1 (6.3)	1 (2.4)
	- ABC	20 (76.9)	14 (87.5)	34 (81.0)
	- ABCC	4 (15.4)	0 (0.0)	4 (9.5)
	- BCC	1 (3.8)	0 (0.0)	1 (2.4)
	- ABBB	0 (0.0)	1 (6.3)	1 (2.4)
	East Asian-type	25 (47.2)	5 (23.8)	30 (40.5)
	- ABD	20 (80.0)	5 (100.0)	25 (83.3)
	- ABBD	5 (20.0)	0 (0.0)	5 (16.7)
	Undetermined	2 (3.7)	0 (0.0)	2 (2.7)
Pre-EPIYA*	No deletion	27 (52.9)	16 (76.2)	43 (59.7)
	6-bp deletion	8 (15.7)	0 (0.0)	8 (11.1)
	18-bp deletion	15 (29.4)	1 (4.8)	16 (22.2)
	39-bp deletion	1 (2.0)	4 (19.0)	5 (6.9)
	Undetermined	2 (3.7)	0 (0.0)	2 (2.7)
*vacA*	s1	53 (100.0)	21 (100.0)	74 (100.0)
	s2	0 (0.0)	0 (0.0)	0 (0.0)
	m1	39 (73.6)	12 (57.1)	51 (68.9)
	m2	14 (26.4)	9 (42.9)	23 (31.1)
	i1	52 (98.1)	21 (100.0)	73 (98.6)
	i2	1 (1.9)	0 (0.0)	1 (1.4)
	d1	40 (75.5)	16 (76.2)	56(75.7)
	d2	13 (24.5)	5 (23.8)	18 (24.3)
	c1	29 (54.7)	12 (57.1)	41 (55.4)
	c2	0 (0.0)	1 (4.8)	1 (1.4)
	c1 and c2	24 (45.3)	8 (38.1)	32 (43.2)
*hrgA*	positive	38 (71.7)	16 (76.2)	54 (73.0)
	negative	15 (28.3)	5 (23.8)	20 (27.0)
*jhp0562/β-gal(1*,*3)T*	*jhp0562*-positive	27 (50.9)	6 (28.6)	33 (44.6)
	*β-gal(1*,*3)T-*positive	6 (11.3)	4 (19.0)	10 (13.5)
	*jhp0562/β-gal(1*,*3)T-*positive	20 (37.7)	11 (52.4)	31 (41.9)

* P <0.05, compared between regions by Chi-square test

**Fig 3:**
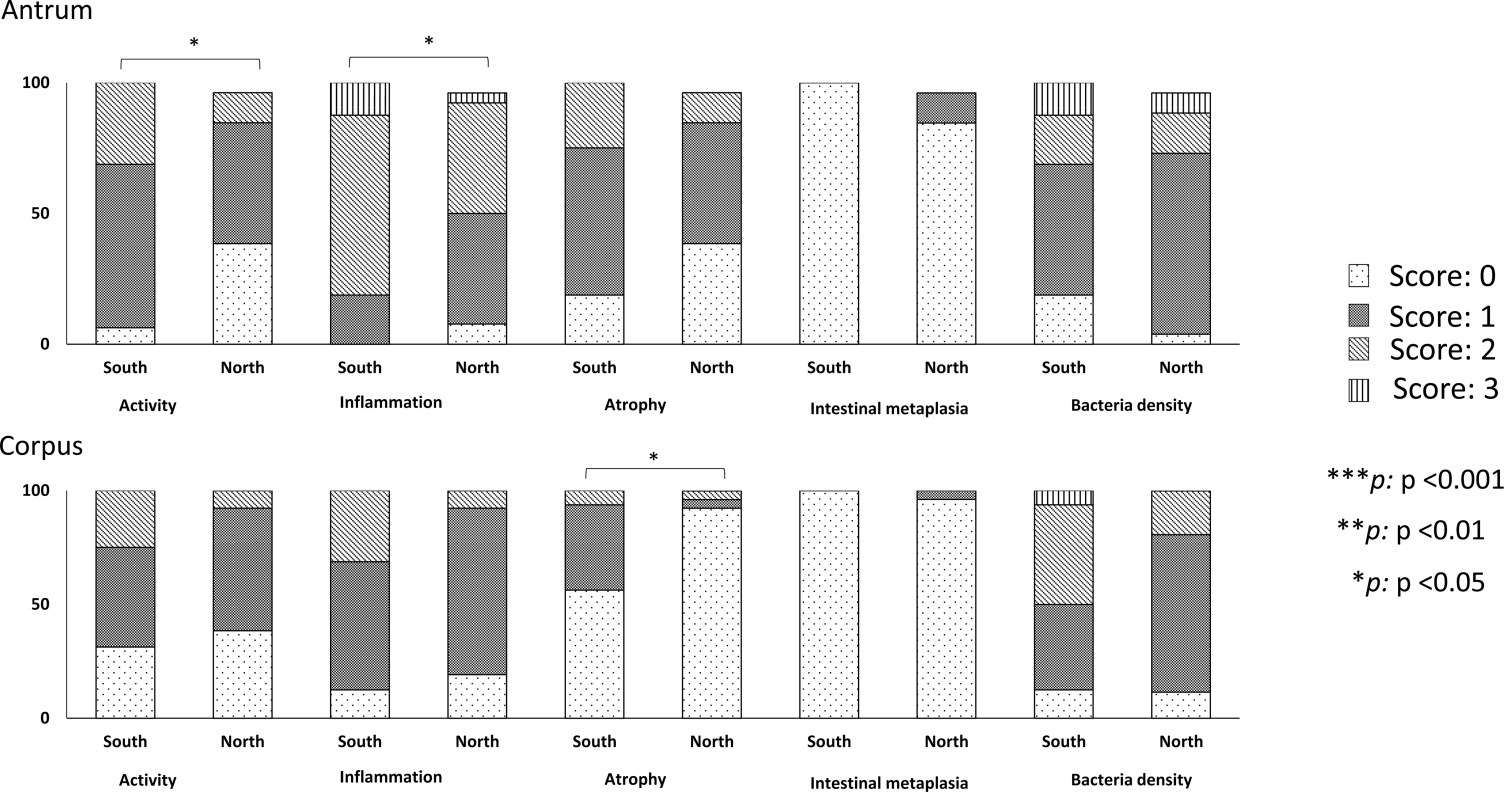
Histological scores of *H*. *pylori* carrying Western-type *cagA* by region. Scores for antrum activity and inflammation, in addition to atrophy in the corpus were significantly higher in patients who were from the South (n = 16) than in those who were from the North (n = 26)(P <0.05, Mann-Whitney U test).

**Fig 4:**
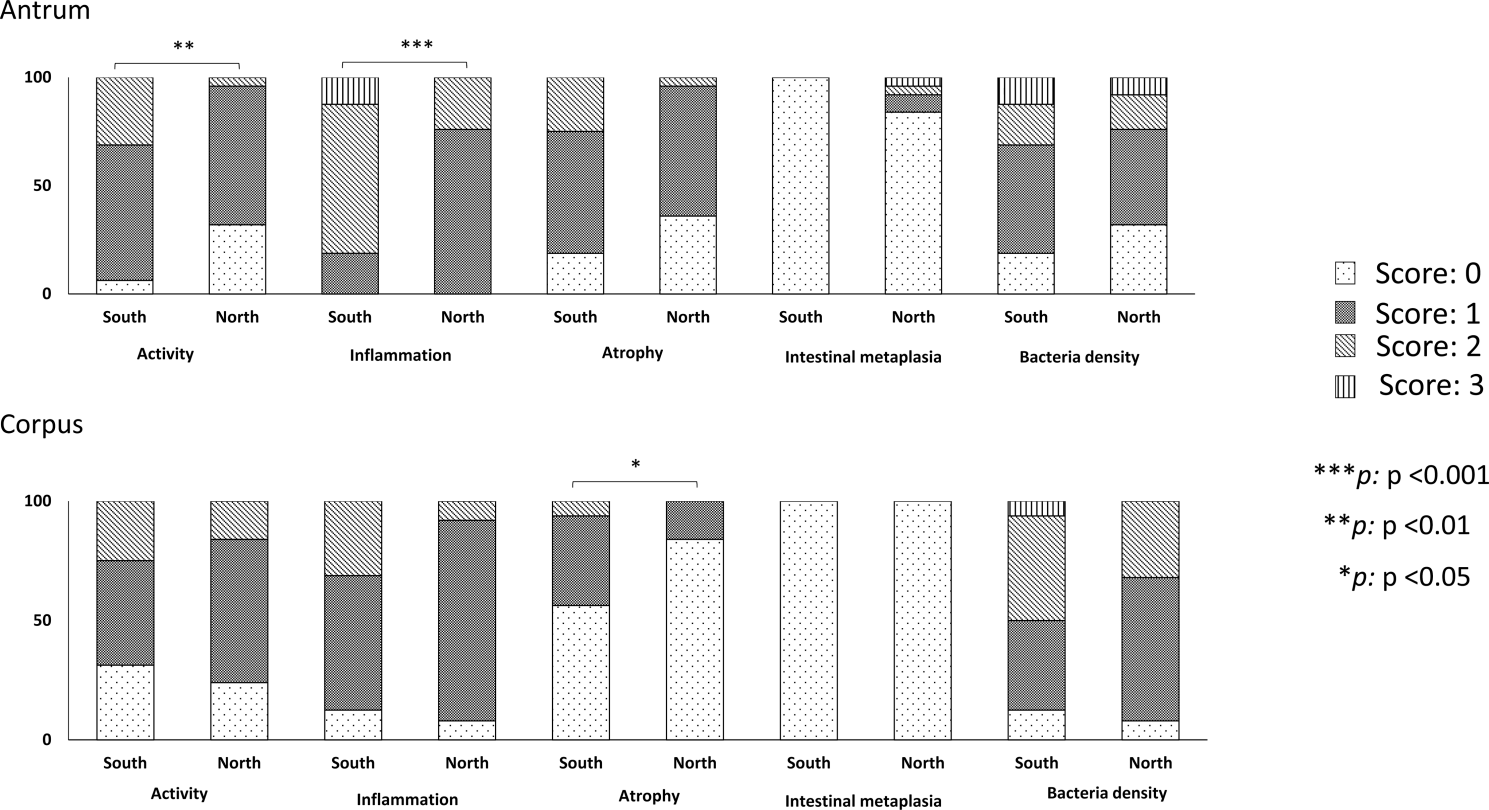
The comparison of histological scores between *H*. *pylori* Western-type *cagA* strains isolated from the South and *H*. *pylori* East Asian-type *cagA* strains isolated from the North. Scores for activity and inflammation in the antrum and atrophy in the corpus of the Western-type *cagA* Southern isolate (n = 16) were significantly higher than those of the East Asian-type *cagA* strains isolated from the North (n = 25)(P <0.05, Mann-Whitney U test).

Surprisingly, patients infected with strains carrying Western-type *cagA* had significantly higher inflammation scores than did patients infected with East Asian-type *cagA* (Table 4). This result was inconsistent with the current consensus. This statistical difference was still apparent, even after using multivariate analysis that adjusted for age, sex, location, and birthplace (OR = 3.98, 95% CI = 1.18 to 13.48, P = 0.026). The distribution of the *cagA* genotype by region is shown in Table 5. The South had a greater proportion of Western-type *cagA* than did the North (76.2%, 16/21 vs. 49.1%, 26/53, P = 0.05, Table 5). There was difference of the distribution of pre-EPIYA among both regions (P = 0.002). There was no difference in histological scores between individuals infected with strains carrying Western-type *cagA* and East Asian-type *cagA* within the same region. However, among patients infected with Western-type *cagA* strains, patients living in the South had significantly higher activity, inflammation scores in the antrum, and atrophy scores in the corpus than did patients in the North (Table 4, Fig 3). There was no such difference among patients infected with East Asian-type *cagA* strains between these two regions. We also analyzed the histological scores between Western-type *cagA* strains from patients living in the South and East Asian-type *cagA* strains from patients living in the North to confirm whether *H*. *pylori* strains from the South were more virulent than were strains from the North (Fig 4). Surprisingly, patients infected with Western-type *cagA* strains from the South had significantly higher activity, inflammation scores in the antrum, and atrophy scores in the corpus than did patients who were infected with East Asian-type *cagA* from the North (1.25 [1] vs. 0.72 [1], P = 0.007, 1.93 [2] vs. 1.24 [1], P < 0.001 and 0.5 [0] vs. 0.16 [0], P = 0.046, respectively). We assume that *H*. *pylori* strains from the South were more virulent than those from the North.

We further analyzed *cagA* in detail, including the pre-EPIYA motif, the EPIYA motif, and the CM motif. Sequence analyses of pre-EPIYA are shown in Table 5. The no-deletion type (typically observed in Western countries) was the predominant type. All Western-type *cagA* strains had the no-deletion type, while East Asian-type *cagA* strains had either the no-deletion (4/30, 13.3%), 6-bp (7/30, 23.3%), 18-bp (14/30, 46.7%), or 39-bp deletion type (5/30, 16.7%). The EPIYA motifs in these strains were also evaluated (Table 6). In total, 224 EPIYA motifs were found in the 72 CagA proteins. The most common 5-amino acid EPIYA motif was EPIYA (200/224, 89.2%), in agreement with our previous studies in Japan and with 4,534 CagA protein sequences from three data sources [48, 49]. The proportion of strains with EPIYT in the North was significantly higher than that in the South (19/160 [11.9%] vs. 0/63 [0.0%], P = 0.001). Importantly, all Western-type *cagA* strains from the South had the EPIYA sequence at the B motif, which was reported to be more highly associated with gastric cancer than was the EPIYT sequence [20].

**Table 6:** EPIYA and EPIYA-like sequence type.

Region	All motif	No. (%)	A motif	No. (%)	B motif	No. (%)	C or D motif	No. (%)
North	EPIYA	138 (86.3)	EPIYA	49 (98.0)	EPIYA	36 (64.3)	EPIYA	53 (98.1)
	EPIYT*	19 (11.9)	EPIYT	1 (2.0)	EPIYT	18 (32.1)	ELIYA	1 (1.9)
	ELIYA	1 (0.6)			EPVYA	1 (1.8)		
	EPVYA	1 (0.6)			ESIYT	1 (1.8)		
	ESIYT	1 (0.6)						
Total		160		50		56		54
South	EPIYA	61 (96.8)	EPIYA	21 (100.0)	EPIYA	22 (91.6)	EPIYA	19 (100.0)
	EPIYD	1 (1.6)			EPIYD	1 (4.2)		
	ESIYA	1 (1.6)			ESIYA	1 (4.2)		
Total		63		21		24		19
All	EPIYA	200 (89.3)	EPIYA	70 (98.6)	EPIYA	58 (72.5)	EPIYA	72 (98.6)
	EPIYT	19 (8.5)	EPIYT	1 (1.4)	EPIYT	18 (22.5)	ELIYA	1 (1.4)
	ELIYA	1 (0.44)			ESIYA	1 (1.25)		
	ESIYA	1 (0.44)			ESIYT	1 (1.25)		
	ESIYT	1 (0.44)			EPIYD	1 (1.25)		
	EPIYD	1 (0.44)			EPVYA	1 (1.25)		
	EPVYA	1 (0.44)						
Total		224		71		80		73

*P = 0.001 compared between regions by Chi-square test

**Fig 5:**
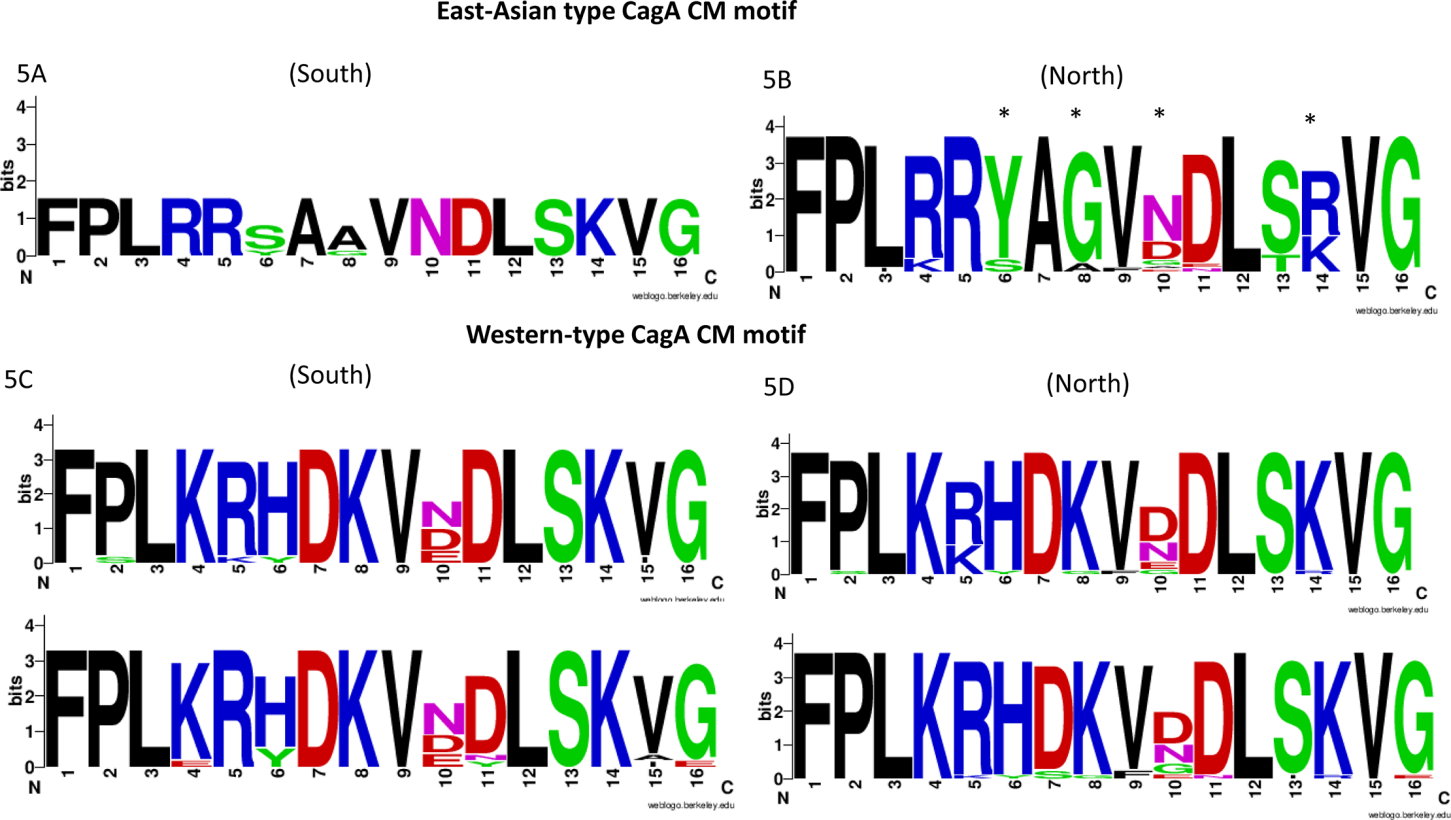
CM motif of East Asian-type and Western-type CagA from Southern and Northern *H*. *pylori* strains. Sequence logos were determined using the program WebLogo, v3. The CM motif was obtained from 5 and 25 strains of East Asian-type CagA from the South and North, respectively (5A and 5B); the star above the amino acid represents a high diversity of amino acids, which are different from the typical sequence. The Western-type CagA CM motifs were obtained from 14 and 25 strains from the South and North, respectively (5C and 5D).

The CM motif is the 16-amino acid sequence that is present in the C-terminal EPIYA-C or EPIYA-D segment. The conserved amino acid sequence motif is FPL*X*R*XXX*V*X*DLSKVG [22]. The typical Western-type *cagA* CM motif located within the EPIYA-C segments and/or immediately distal to the last repeat of the EPIYA-C segment contains the peptide sequence FPLKRHDKVDDLSKVG, while typical East Asian-type *cagA* carries only one CM motif (FPLRRSAAVNDLSKVG) [22, 48]. The CM motif sequences of strains from Thailand are shown in Table 7 and Fig 5. All 30 East Asian-type *cagA* strains and 39 of the Western-type *cagA* strains (AB, ABB, and ABBB were excluded because they only contained one or no CM motif) were included. Among East Asian-type *cagA* strains, 80% (4/5) of strains from the South had CM motif sequences typically observed in East Asian strains, while the typical motif sequences were rare among strains from the North (1/25, 4.0%) (Table 7, Fig 5A and 5B). Less than 30% of the 1^st^ and 2^nd^ motif sequences of Western-type *cagA* contained typical Western-type CM motif sequences (11/39 (28.2%) and 8/39 (20.5%), respectively). There were reports that the sequence patterns of pre-EPIYA, the CM motif, and EPIYA sequences were related to pathophysiological activities of CagA [19, 20, 22]; however, we could not find an association between histological scores and their sequences patterns.

**Table 7:** The CM motif of Western-type and East Asian-type *cagA*.

*cagA* genotype		Sequence	North	South	Total
East Asian-type *cagA*	1^st^ motif	FPLRRSAAVNDLSKVG	1	4	5
		Others	24	1	25
	Total		25	5	30
Western-type *cagA*	1^st^ motif	FPLKRHDKVDDLSKVG	6	5	11
		Others	19	9	28
	Total		25	14	39
	2^nd^ motif	FPLKRHDKVDDLSKVG	6	2	8
		Others	19	12	31
	Total		25	14	39

All of the *vacA*-carrying strains were of the s1 genotype. As for the m region, the *vacA* m1 genotype was predominant (68.9%) (Table 5). i1, c1, and d1 were the predominant genotypes at their respective regions (98.6%, 55.4%, and 75.7%). All strains with the s1m1 genotypes (68.9%) also contained the i1 genotype. After examining all the *vacA* subtypes, it was determined that the s1m1i1c1d1 and s1m1i1c1c2d1 genotypes were predominant (32.4% and 24.3%, respectively). However, there was no association between *vacA* subtypes and histological scores or regions. The majority of isolates carried the *hrgA* gene (73.0%). *jhp0562*-positive/*β-(1*,*3)galT*-negative and *jhp0562*/*β-(1*,*3)galT*-double positive were the predominant types (44.6% and 41.9%, respectively). There was no association between the status of either gene and histological scores or regions.

### IL-8 levels produced from AGS cells infected with *H*. *pylori*

AGS cells were co-cultured with various *H*. *pylori* strains. As shown in S3 Fig, the South *H*. *pylori* strains had IL-8 production slightly higher than the North strains, but the difference were not statistically significant (average IL-8 levels: 2,371 vs. 2,246 pg/mL, P = 0.76). In contrast with our expectation, within the South strains; the East-Asian-type *cagA* strains tended to induce higher IL-8 production than Western-type *cagA* strains (2,602 vs. 2,139 pg/mL, P = 0.076). However, there was no such difference among two types of *cagA* strains in the North (2,274 vs. 2,217 pg/mL, P = 0.92).

## Discussion

In this study, we examined the *H*. *pylori* infection status in the North and South of Thailand. One important finding was that the prevalence of *H*. *pylori* infection was significantly higher in subjects who were living in the North (50.4%) than in those living in the South (17.6%), which might explain the variation in gastric cancer risk in Thailand, where the incidence of gastric cancer is approximately three times higher in the North than in the South [33].

However, our findings showed that patients in the South had more severe histological scores than did those in the North among *H*. *pylori*-infected patients. Therefore, we further examined the virulence genotypes of *H*. *pylori*. Surprisingly, histological analysis showed that inflammation scores in the antrum were significantly higher in subjects infected with Western-type *cagA* strains than the scores were in patients infected with East Asian-type *cagA* strains, even after being adjusted for age, sex, location, and birthplace. Moreover, our findings showed that Western-type *cagA* strains from the South could induce more severe activity, inflammation in the antrum, and atrophy in the corpus than could either of the *cagA*-genotype strains from the North. These results suggested that *H*. *pylori*-infected patients from the South might have a higher risk of developing gastric cancer. There are several possible explanations for our unexpected data. First, *H*. *pylori* from the South has a higher proportion of strains with EPIYA at the B motif than does *H*. *pylori* from the North; this difference might be associated with its virulence. An analysis of Western-type *cagA* revealed evidence that strains with EPIYA sequences of the EPIYA-B motif were significantly more associated with gastric cancer than were those with EPIYT sequences [20]. Secondly, strains in the South might have unknown virulence gene(s) other than *cagA/vacA*. We only examined several putative virulence factors. Unexpected data in this study are not unique, and our previous study showed that Western-type *cagA* in Nepalese strains was more virulent than was East Asian-type *cagA* [40]. In addition, our previous study using IHC showed that most of the Mongolian population that was infected with *H*. *pylori* did not react with an anti-East Asian-type CagA-specific antibody [50], which has immunoreactivity only against the East Asian-type CagA strains and not against Western-type CagA strains [44]. These data suggested that in Mongolia, where the incidence of gastric cancer death is the highest worldwide, most strains were either *cagA*-negative or Western-type *cagA*. Therefore, it will be impossible to explain the virulence only by the genotypes of *cagA* alone or even by the combination of *cagA* with well-known virulence factors such as *vacA*. Since *H*. *pylori* contains approximately 1,500 genes, other unknown virulence factors of *H*. *pylori* might play important roles in its pathogenesis in Thailand. Further studies are necessary to search for new virulence genes using next-generation sequencing. Thirdly, factors other than bacterial factors play important roles for promoting pathological differences between the North and the South.We examined the ability to induce IL-8 from gastric cells and there was no definite relationship on IL-8 production between the *cagA* genotypes and the location. However, since IL-8 is only one of the pathological markers and further studies will be necessary by measuring other pathological markers to confirm the roles of bacterial factors on the pathogenesis. Finally, the most possible explanation is that subjects from the South may have increased gastric cancer risk after *H*. *pylori* infection because of an interaction between host genotypes and bacterial virulence. For example, the highest gastric cancer risk in a Western country is in individuals with a combination of the high-expression type of interleukin-1 alleles and colonization with *vacA* s1-type *H*. *pylori*, probably due to amplification of mucosal inflammatory response and inhibition of acid production [51].

Also recent studies show a mismatch of host (individual) ancestry versus bacterial (*H*. *pylori*) ancestry can lead to more severe histology. A study in Colombia showed that African *H*. *pylori* ancestry was relatively benign in humans of African ancestry but was deleterious in individuals with substantial Amerindian ancestry, suggesting that coevolution likely modulated disease risk [52]. In this study, we did not examine host factors in detail (e.g., single nucleotides polymorphisms of inflammatory cytokines), and these factors might explain the different rates of incidence of gastric cancer among *H*. *pylori-*infected patients in the North and South.

Here, we showed that several socio-demographic factors were associated with an increasing prevalence of *H*. *pylori* infection. A subject with a low income was significantly more likely to be infected with *H*. *pylori* than those with a high economic status. Additionally, the North had a greater number of subjects with low economic status than did the South. These data are in agreement with the fact that with the exception of Bangkok the total monthly income per household in 2013 in the North (19,267 THB, which is approximately 849.9 USD in February 2017) was lower than the income in the South (27,504 THB) (data available at Statistical yearbook Thailand 2013, http://web.nso.go.th). Age, birthplace in the North, unboiling water consumption, being a farmer, and greater alcohol consumption were risk factors for greater prevalence of *H*. *pylori* infection in Thai populations, although the region is the most important factor, based on multivariate analysis. Interestingly, the age groups of 40–49 and 50–59 had the highest risk for *H*. *pylori* infection; however the prevalence of *H*. *pylori* infection was decreased in patients over 60 which might not be associated with the timing of the improvement of sanitation in Thailand. Further study will be necessary for clarify the age related risk for *H*. *pylori* infection taking the previous infection in elder ages into account. Some other factors might also become risk factors for gastric cancer after further examination. Our previous study showed that Chinese individuals living in Thailand have a high gastric cancer risk [16]; however, individuals of Chinese ethnicity were not examined in this study. Although there was no association between fermented food consumption and regional differences, a previous study reported that a variety of dietary habits appear to greatly influence pan-gastritis frequency in Thailand, irrespective of *H*. *pylori* infection rate [53]. For example, the North is a mountainous area, and individuals consume fermented, salty sauces with hot and spicy foods daily, which are dietary habits that induce gastritis; on the other hand, people in the South, a coastal region, have seafood, beans, fresh vegetables, and fruits as their common dishes [53]. Similarly, gastric cancer is extremely common in individuals from the mountainous regions of Central and South America and is associated with dietary habits [54].

This study has several limitation. First, study population in this study came from dyspeptic patients; therefore the specifically was not the general population. However, our small number of preliminary data for volunteers without dyspeptic symptoms in each region showed the similar prevalence of *H*. *pylori* infection to that of dyspeptic patients (Subsomwong P et al., unpublished data). Further population-based study using the large number of participants will be necessary to confirm our current data. Finally, we only obtained samples from two cities in the North and one city in the South. Therefore, our results might not be generalizable across Thailand. Further investigation is necessary to obtain samples from other cities in both regions, as well as from other regions throughout the country. These studies are now in progress.

## Conclusion

The prevalence of *H*. *pylori* infection in Thailand is associated with the incidence of gastric cancer. Importantly, Western-type *cagA* strains were even more virulent in patients from the South than were strains with either *cagA* genotype in patients from the North. Therefore, a more careful follow-up for patients infected with *H*. *pylori* in the South is required, even if these patients are infected with Western-type *cagA* strains.

## Supporting information

S1 Fig Histological scores by region. Patients who were living in the North (n = 137) had higher antrum histological scores than did those who were living in the South (n = 136); this trend was the same for activity and inflammation in the corpus (P <0.05, Mann-Whitney U test).
(TIF)

S2 Fig Histological scores corresponding to *H*. *pylori* status. For the antrum and corpus of the *H*. *pylori*-infected group (n = 93), all histological scores aside of intestinal metaplasia were significantly higher than they were for the *H*. *pylori*-uninfected group (n = 180, P <0.05, Mann-Whitney U test).
(TIF)

S3 Fig IL-8 concentrations produced by AGS cells infected with *H*. *pylori* strains for 24 hours. The strains were divided by regions (the North and the South) and by *cagA* and regions (Western-type and East-Asian-type *cagA*). Bars represent means of IL-8 concentration (three independent experiment) in duplicates ± SD.
(TIF)

S1 Table The list of primers used for genotyping *H*. *pylori* virulence genes. (DOCX)

S2 Table Association of demographics and sanitation levels with *H*. *pylori* infection status. (DOCX)

S3 Table Histological scores by region and *H*. *pylori* status. (DOCX)

## Abbreviations

IHC: immunohistochemistry
OLGA: Operative Link on Gastritis Assessment system
bp: base pair
