# Supplementary figures and images for: Prevalence, risk factors, and virulence genes of Helicobacter pylori among dyspeptic patients in two different gastric cancer risk regions of Thailand
Source: PLoS One. 2017 Oct 30;12(10):e0187113. doi: 10.1371/journal.pone.0187113 (PMC5662176; doi:10.1371/journal.pone.0187113)

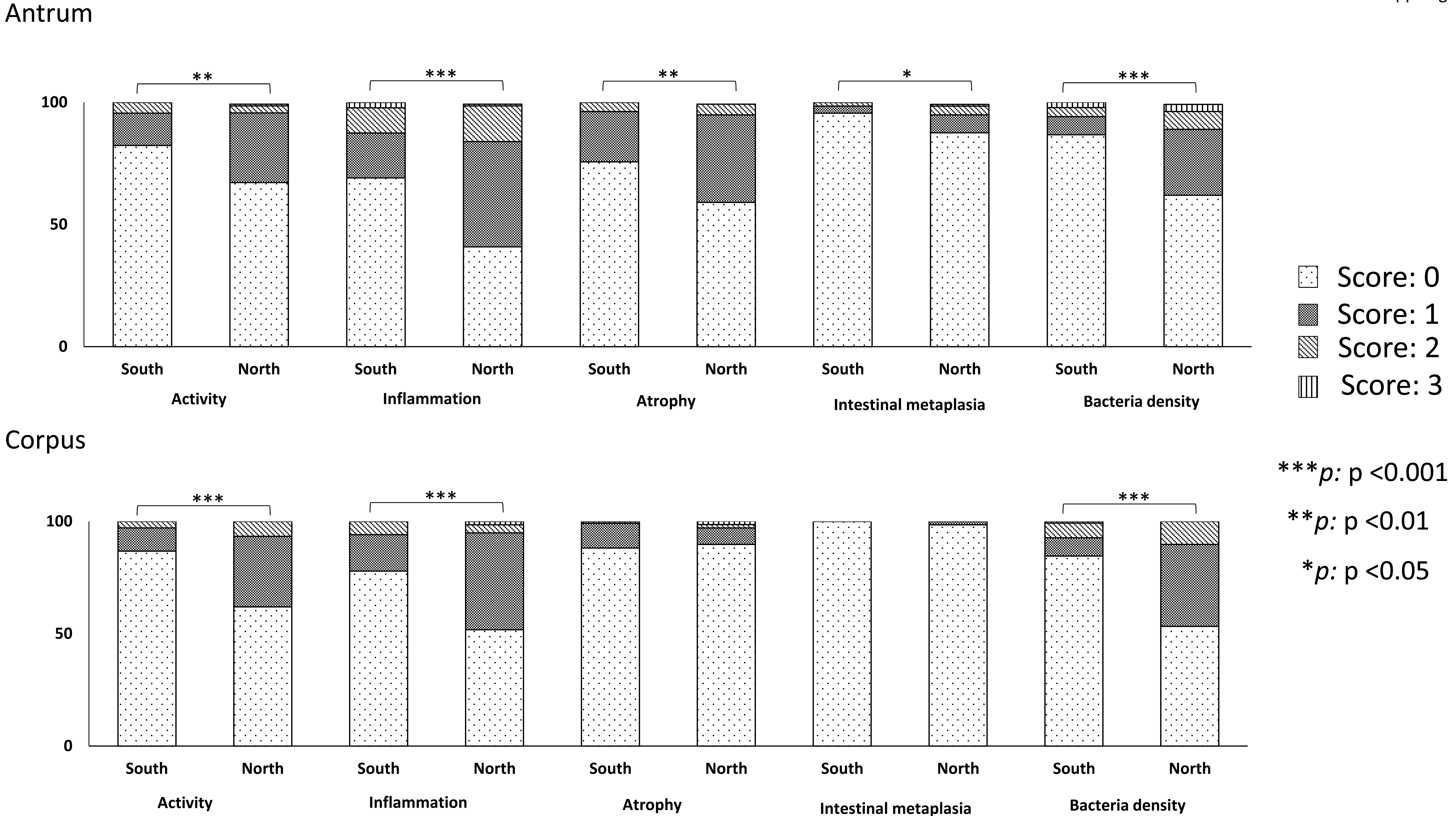

Supplement: S1 Fig — Patients who were living in the North (n = 137) had higher antrum histological scores than did those who were living in the South (n = 136); this trend was the same for activity and inflammation in the corpus (P <0.05, Mann-Whitney U test). (TIF) [file pone.0187113.s001.tif]

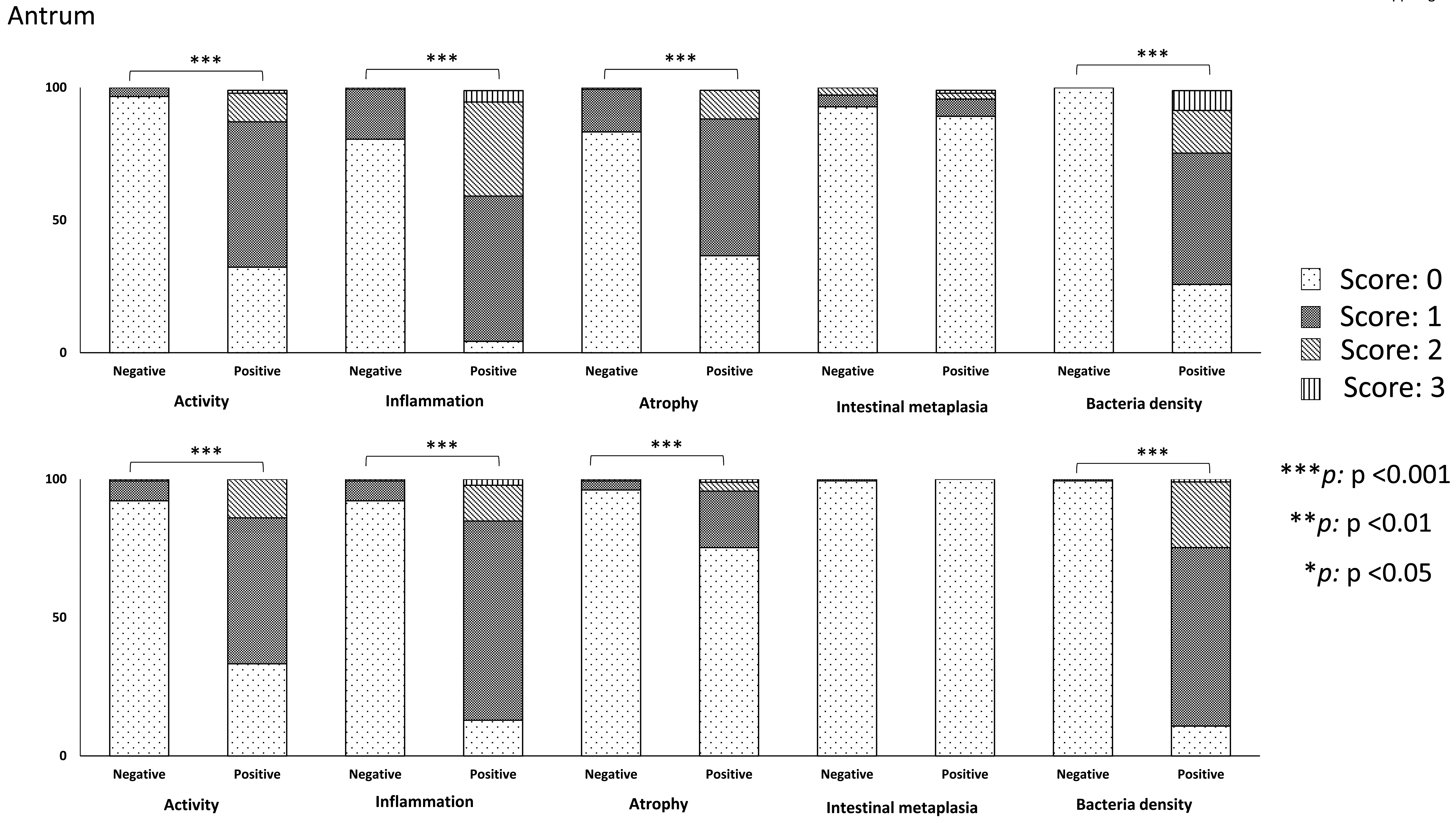

Supplement: S2 Fig — For the antrum and corpus of the H. pylori-infected group (n = 93), all histological scores aside of intestinal metaplasia were significantly higher than they were for the H. pylori-uninfected group (n = 180, P <0.05, Mann-Whitney U test). (TIF) [file pone.0187113.s002.tif]

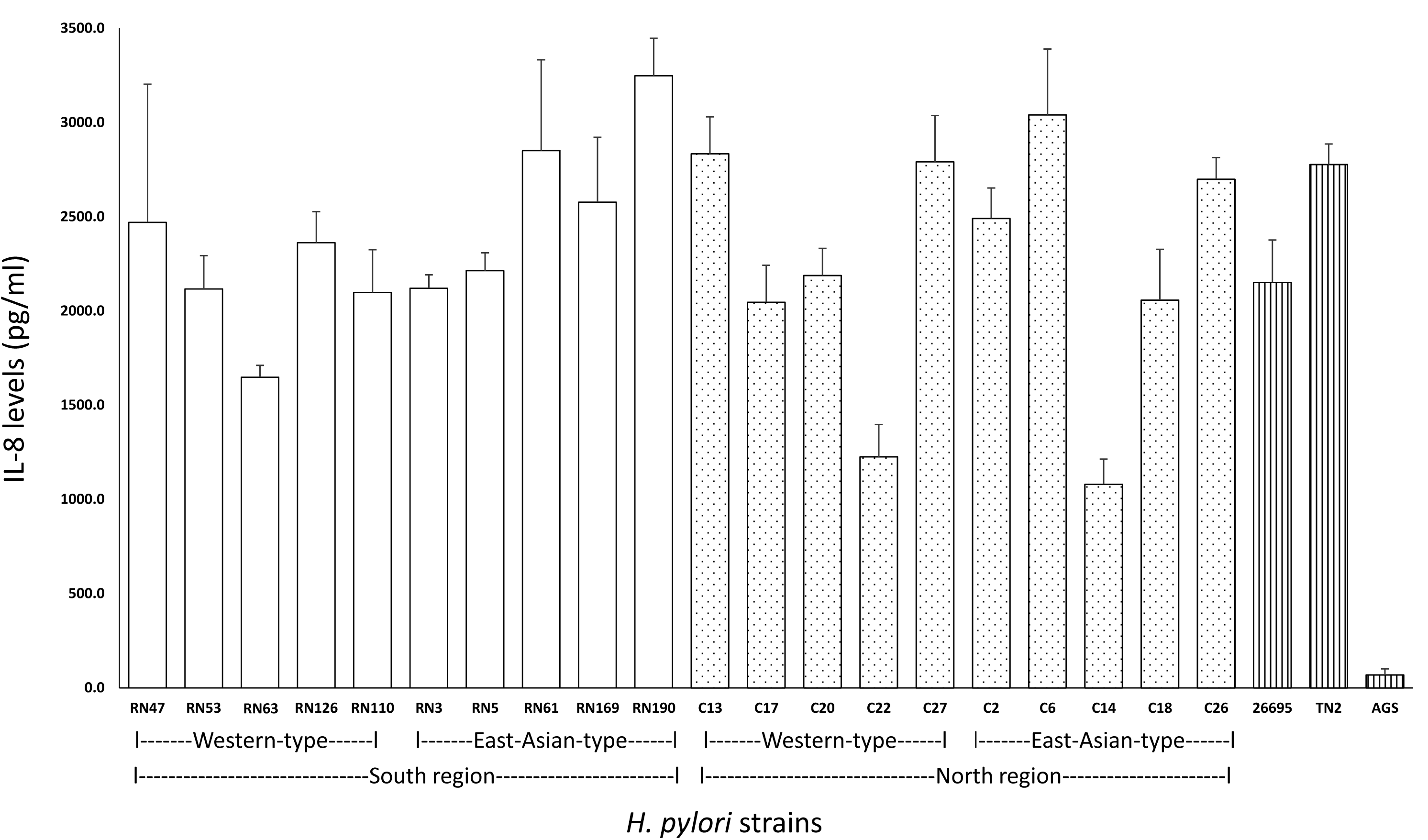

Supplement: S3 Fig — The strains were divided by regions (the North and the South) and by cagA and regions (Western-type and East-Asian-type cagA). Bars represent means of IL-8 concentration (three independent experiment) in duplicates ± SD. (TIF) [file pone.0187113.s003.tif]
