# Supplementary material for: Prevalence, risk factors, and virulence genes of Helicobacter pylori among dyspeptic patients in two different gastric cancer risk regions of Thailand
Source: PLoS One. 2017 Oct 30;12(10):e0187113. doi: 10.1371/journal.pone.0187113 (PMC5662176; doi:10.1371/journal.pone.0187113)
Supplement: S1 Table — (DOCX) [file pone.0187113.s004.docx]

**Suppl. table 1** The list of primer for genotyping of *H. pylori* virulence genes

| Gene | Primer sequence (5’-3’) |
| --- | --- |
| *cagA* | *cagA* OMF: AGC AAA AAG CGA CCT TGA AA  *cagA* OMR: ATT CAC GAG CTT CAG CCA CT |
| *vacA*  *vacA* s region  *vacA* m region  *vacA* i region  *vacA* d region  *vacA* c region | VA1F: ATG GAA ATA CAA CAA ACA CAC  VA1R: CTG CTT GAA TGC GCC AAA C  VAGF: CAA TCT GTC CAA TCA AGC GAG  VAGR: GCG TCT AAA TAA TTC CAA GG  VacF1: GTT GGG ATT GGG GGA ATG CCG  C1R: TTA ATT TAACGC TGT TTG AAG  C2R: GAT CAA CGC TCT GAT TTG A  VAS5F: ACT AAT ATT GGC ACA CTG GAT TTG  VAGFR: CTC GCT TGA TTG GAC AGA TTG  C1F: ATC ATY SGT TAT GRH AAT GTT TCT  Rnd: TTA TGC TCT AAA CTG GCT A  C2F: ATT ATA ATT TAG GAG TGC AAG G  Rnd: TTA TGC TCT AAA CTG GCT A |
| *hrgA* | hrgA-F: TCTCGTGAAAGAGAATTTCC  hrgA-R: TAAGTGTGGGTATATCAATC |
| *jhp0562/B-gal(1,3)T* | jhp0562F: TGA AAA GCC CTT TTG ATT TTG  jhp0562R: GCT GTA GTG GCC ACA TAC ACG |
