# Supplementary material for: Prevalence, risk factors, and virulence genes of Helicobacter pylori among dyspeptic patients in two different gastric cancer risk regions of Thailand
Source: PLoS One. 2017 Oct 30;12(10):e0187113. doi: 10.1371/journal.pone.0187113 (PMC5662176; doi:10.1371/journal.pone.0187113)
Supplement: S2 Table — (DOCX) [file pone.0187113.s005.docx]

**Suppl. table 2** Association of demographics and sanitation levels with *H. pylori* infection status

| Variable | *H. pylori*-positive/total number (%) | Crude OR | 95% CI for OR | P |
| --- | --- | --- | --- | --- |
| Age |  |  |  |  |
| <29 | 6/19 (31.6) | 1.66 | 0.42-6.64 | 0.47 |
| 30-39 | 5/23 (21.7) | 1.00 |  |  |
| 40-49 | 23/53 (43.4) | 2.76 | 0.89-8.54 | 0.08 |
| 50-59 | 34/83 (41.0) | 2.50 | 0.85-7.38 | 0.10 |
| >60 | 25/95 (26.3) | 1.29 | 0.43-3.83 | 0.65 |
| Gender |  |  |  |  |
| Male | 37/95 (38.9) | 1.39 | 0.83-2.34 | 0.22 |
| Female | 56/178 (31.5) | 1.00 |  |  |
| Ethnicity |  |  |  |  |
| Thai | 83/246 (33.7) | 1.53 | 0.16-14.91 | 0.72 |
| Lao | 5/10 (50.0) | 3.00 | 0.23-39.61 | 0.41 |
| Burmese | 0/2 (0.0) | 0.00 | 0.00 | 0.99 |
| Hmong | 2/4 (50.0) | 3.00 | 0.15-59.89 | 0.50 |
| Lue | 1/4 (25.0) | 1.00 |  |  |
| Others | 2/5 (40.0) | 2.00 | 0.11-35.81 | 0.64 |
| Occupation |  |  |  |  |
| Agriculture | 37/81 (45.7) | 3.92 | 1.47-10.5 | 0.006 |
| Employee | 12/36 (33.3) | 2.24 | 0.73-6.86 | 0.16 |
| Government job | 6/35 (17.1) | 1.00 |  |  |
| Merchant | 7/26 (26.9) | 1.72 | 0.50-5.92 | 0.39 |
| Other | 15/41 (36.6) | 1.97 | 0.68-5.66 | 0.21 |
| Unemployed | 16/54 (29.6) | 2.69 | 0.91-7.98 | 0.07 |
| Marital status |  |  |  |  |
| Divorced | 7/19 (36.8) | 1.82 | 0.54-6.21 | 0.34 |
| Married | 77/220 (35.0) | 1.68 | 0.72-3.91 | 0.23 |
| Single | 8/33 (24.2) | 1.00 |  |  |
| Body Mass Index |  |  |  |  |
| <18.5 | 8/18 (44.4) | 2.04 | 0.52-8.00 | 0.31 |
| 18.5-24.9 | 59/168 (35.1) | 1.53 | 0.53-4.46 | 0.44 |
| 25-29.9 | 20/67 (29.9) | 1.19 | 0.38-3.75 | 0.77 |
| >30 | 5/19 (26.3) | 1.00 |  |  |
| Fried food |  |  |  |  |
| No | 44/127 (34.6) | 1.05 | 0.64-1.73 | 0.85 |
| Yes | 49/146 (33.6) | 1.00 |  |  |
| Fermented food |  |  |  |  |
| No | 56/176 (31.8) | 1.00 |  |  |
| Yes | 37/97 (38.1) | 1.32 | 0.79-2.22 | 0.29 |
| Smokers |  |  |  |  |
| No | 61/199 (30.7%) | 1.00 |  |  |
| Yes | 32/74 (43.2%) | 1.72 | 1.00-2.99 | 0.052 |

P <0.05, Chi-square, estimate risk by OR and 95% CI
