# Supplementary material for: Prevalence, risk factors, and virulence genes of Helicobacter pylori among dyspeptic patients in two different gastric cancer risk regions of Thailand
Source: PLoS One. 2017 Oct 30;12(10):e0187113. doi: 10.1371/journal.pone.0187113 (PMC5662176; doi:10.1371/journal.pone.0187113)
Supplement: S3 Table — (DOCX) [file pone.0187113.s006.docx]

**Suppl. Table 3. Histological scores according region and *H. pylori* status**

| Histological parameters | Region | | p | *H. pylori* | | p |
| --- | --- | --- | --- | --- | --- | --- |
|  | North | South |  | Positive | Negative |  |
| N | 137 | 136 |  | 93 | 180 |  |
| **Antrum** |  |  |  |  |  |  |
| Activity | 0.37 (0) | 0.22 (0) | 0.008 | 0.80 (1) | 0.03 (0) | <0.001 |
| Inflammation | 0.75 (1) | 0.46 (0) | <0.001 | 1.41 (1) | 0.19 (0) | <0.001 |
| Atrophy | 0.45 (0) | 0.28 (0) | 0.006 | 0.74 (1) | 0.17 (0) | <0.001 |
| Intestinal metaplasia | 0.17 (0) | 0.06 (0) | 0.026 | 0.14 (0) | 0.10 (0) | 0.466 |
| Bacterial density | 0.51 (0) | 0.21 (0) | <0.001 | 1.07 (1) | 0.00 (0) | <0.001 |
| **Corpus** |  |  |  |  |  |  |
| Activity | 0.45 (0) | 0.16 (0) | <0.001 | 0.83 (1) | 0.03 (0) | <0.001 |
| Inflammation | 0.55 (0) | 0.28 (0) | <0.001 | 1.05 (1) | 0.08 (0) | <0.001 |
| Atrophy | 0.15 (0) | 0.13 (0) | 0.739 | 0.30 (0) | 0.05 (0) | <0.001 |
| Intestinal metaplasia | 0.15 (0) | 0.00 (0) | 0.319 | 0.01 (0) | 0.01 (0) | 0.472 |
| Bacterial density | 0.57 (0) | 0.05 (0) | <0.001 | 1.18 (1) | 0.00 (0) | <0.001 |
| **OLGA score** | 0.51 (0) | 0.29 (0) | 0.002 | 0.83 (1) | 0.19 (0) | <0.001 |

P <0.05, Mann-Whitney U test
